# Medical Corporations

**Published:** 1836-01

**Authors:** 


### BRITISH MEDICAL CORPORATIONS.

No. 1. *Degrees in Medicine* (M.B., M.D.,) *conferred in the British Universities during the last Ten Years.*

| Universities. | 1826 | 1827 | 1828 | 1829 | 1830 | 1831 | 1832 | 1833 | 1834 | 1835 | Total |
|---|---|---|---|---|---|---|---|---|---|---|---|
| Oxford - - | 3 | 4 | 1 | 4 | 4 | 2 | 1 | 7 | 6 | 3 | 35 |
| Cambridge - | 10 | 7 | 13 | 13 | 14 | 13 | 13 | 12 | 11 | 12 | 118 |
| Edinburgh - - | 119 | 160 | 93 | 99 | 107 | 120 | 112 | 110 | 110 | 117 | 1147 |
| Glasgow - - | 26 | 18 | 41 | 33 | 37 | 29 | 48 | 59 | 78 | 79 | 448 |
| Total in ten years | 158 | 189 | 148 | 149 | 162 | 164 | 174 | 188 | 205 | 211 | 1748 |

*Note* 1.—In all the Universities, except Oxford and Cambridge, the degrees are exclusively those of M.D. The following are the respective numbers of M.B. and M.D. degrees in the two last, during the ten years comprehended in the list:

| | | M.B. | M.D. |
|---|---|---|---|
| Oxford | - | 24 | 11 |
| Cambridge | - | 82 | 36 |

In the above list of Oxford graduations, are not included the degrees of M.D. by diploma, granted in July, 1835, at the meeting of the Provincial Medical and Surgical Association, to Dr. Abercrombie, of Edinburgh, and Dr. Prichard, of Bristol; the only instances of diploma M.D. degrees during the last fifty years, with the single exception of Dr. Jenner.

*Note* 2.—We have not been able to ascertain the number of degrees in medicine conferred in the university of Dublin, nor whether any or what number of degrees in medicine have been conferred at King's College, Aberdeen.

*Note* 3.—No degree in medicine has been conferred in Marischal College, Aberdeen, since the introduction of the new regulations, about eight years ago, except one honorary degree; and the reason of this seems to be the greater strictness of the regulations in that, than in some other of the Scottish Universities. At Marischal College the candidate, previously to examination, must be twenty-five years of age, and M.A. of some University. The medical students, at this time at Marischal College, are upwards of a hundred; but, latterly, all who have intended to graduate have gone to Edinburgh.

*Note* 4.—In the year 1833, the Senatus Academicus of the University of St. Andrew's passed new regulations for granting medical degrees, which, although not so strict as those of Marischal College, render necessary, on the part of the candidate, a full course of previous medical study, during four years, and an examination before the Professor of medicine and five physicians and surgeons, denominated *Conjunct Examinators.* The age of the candidate is only required to be twenty-one. Since these regulations have been in force, twenty-one gentlemen have passed their examinations, and obtained the degree of M.D.

No. 2. *Admissions into the Royal College of Physicians, London, during Ten Years.*

| | 1824 | 1825 | 1826 | 1827 | 1828 | 1829 | 1830 | 1831 | 1832 | 1833 | Total |
|---|---|---|---|---|---|---|---|---|---|---|---|
| Fellows - | 2 | 5 | 5 | 5 | 4 | 1 | 3 | 9 | 6 | 2 | 42 |
| Licentiates | 20 | 16 | 14 | 11 | 9 | 7 | 13 | 7 | 6 | 9 | 112 |
| Total | 22 | 21 | 19 | 16 | 13 | 8 | 16 | 16 | 12 | 11 | 154 |

No. 3. *Surgical Diplomas granted by the British Colleges during Ten Years.*

| Colleges. | 1824 | 1825 | 1826 | 1827 | 1828 | 1829 | 1830 | 1831 | 1832 | 1833 | Total |
|---|---|---|---|---|---|---|---|---|---|---|---|
| London - - | 205 | 282 | 302 | 286 | 298 | 340 | 389 | 359 | 267 | 397 | 3125 |
| Edinburgh - - | 130 | 152 | 167 | 187 | 185 | 199 | 187 | 177 | 163 | 169 | 1716 |
| Glasgow - - | 31 | 40 | 23 | 9 | 16 | 20 | 16 | 23 | 18 | 27 | 223 |
| Total in each year | 366 | 474 | 492 | 482 | 499 | 559 | 592 | 559 | 448 | 593 | 5064 |

*Note*—In Glasgow the diplomas are granted by the university: they are termed Degrees of Master in Surgery, and the Graduates, *Masters in Surgery.*

[In our next Number we shall give the Medical and Surgical Degrees conferred in Dublin, and the Licences granted by the Apothecaries' Company, London, during the same years.]

